# Cancer-Oriented Comprehensive Nursing Services in Republic of Korea: Lessons from an Oncologist’s Perspective

**DOI:** 10.3390/medicina59010144

**Published:** 2023-01-11

**Authors:** Suk Hun Ha, Moonho Kim, Hyojin Kim, Boram No, Ara Go, Miso Choi, Seol Lee, Yongchel Ahn

**Affiliations:** 1 Department of Hematology and Oncology, Gangneung Asan Hospital, University of Ulsan College of Medicine, 38 Bangdong-gil, Sacheon-myeon, Gangneung-si 25440, Gangwon-do, Republic of Korea; 2 Department of Nursing, Gangneung Asan Hospital, University of Ulsan College of Medicine, 38 Bangdong-gil, Sacheon-myeon, Gangneung-si 25440, Gangwon-do, Republic of Korea

**Keywords:** cancer, comprehensive nursing service, outcome

## Abstract

*Background and objectives:* As is well known, cancer patients require extensive medical attention as they undergo surgery, chemotherapy, radiotherapy, and supportive care. The importance of high-quality cancer-directed nursing, combined with precision medicine, to maximize their survival outcomes and help them achieve a better quality of life cannot be overemphasized. In this context, we offered a new cancer-oriented comprehensive nursing system to our inpatients and reviewed its clinical outcomes in comparison with those from the preexisting general cancer ward. *Materials and Methods*: From March 2019 to February 2020, a total of 102 cancer patients and 42 nurses were enrolled in this pilot study. We aimed to analyze their performance in three main categories: structure, process, and patient/nurse outcomes. *Results:* First, structural (nurse staffing and environment) upgrades were installed in the cancer-oriented comprehensive nursing ward, including an improved nurse-patient ratio (1:8 in the comprehensive ward as compared with 1:14 in the general ward), wider space between beds (1.5 m versus 1.0 m), fully automatic beds with fall prevention sensors, etc. Second, the nursing process was improved (missed care 0.1 event/month vs. 1.3 event/month). Third, both patient and nurse outcomes showed preferable results in the comprehensive ward. The patient satisfaction level was higher in the comprehensive nursing ward than in the general ward (willing to revisit: 91.7% and 78.4%, respectively; willing to recommend to others: 95.0% and 76.8%, respectively). Pressure ulcers, as a patient safety indicator, were also decreased (0.3 events/month vs. 0.8 events/month). However, the fall incidence was similar in both groups (1.6 events/month vs. 1.5 events/month). In terms of nurse outcomes, turnover intention was stabilized and nurses’ job satisfaction in the comprehensive ward was superior to that of their counterparts. *Conclusions*: Our study was a pilot study to demonstrate that cancer patient-oriented comprehensive nursing services can be helpful in improving the quality of cancer treatment and nurses’ job satisfaction. Continued interest in and efforts to improve nursing care delivery are also crucial in achieving and maintaining the best possible cancer patient care.

## 1. Introduction

In the Republic of Korea, unlike in other developed countries, inpatient nursing care has been shared by the patient’s family members or paid caregivers. For example, family members are allowed to stay in hospital rooms to help with the daily medical care of patients, which enables a relatively small number of medical staff to manage a larger number of patients. We have maintained this individual-patient nursing care system for more than 70 years [1,2]. However, medical problems such as increased opportunistic infections or unexpected falls are more frequently observed when non-medical personnel take part in nursing than in nurse-centered caregiving [3]. In addition, about 40% of inpatients are now cared for by patient-paid caregivers because of the rapid progress of industrialization/urbanization, the increase in the aging population, and the subsequent expansion of the nuclear family. Securing a private caregiver inevitably brings about increased medical expenses for the patients, and it has now become a public healthcare issue.

In an effort to solve this healthcare problem, our government kicked off a pilot project called the “Patient-Sitter Program” in 2006 [4]. The current model of comprehensive nursing care was built to extend beyond the simple concept of co-nursing and was officially initiated with a national insurance subsidy in 2016 [5,6]. This inpatient nursing service features nursing staff reinforcement, along with hospital facility improvements. Registered nurses (RNs) and nursing assistants (NAs) are sufficiently assigned to the comprehensive nursing ward, while patients only need to pay a quarter of the caregiving fee instead of hiring a private caregiver [1]. As of December 2019, this comprehensive nursing system was successfully implemented, with 49,000 beds in 534 institutions [6]. Reports on the performance of this service mainly focused on patient and nurse satisfaction from the nurses’ perspectives. Studies generally showed that the risk of infection and accidents was reduced in this service, while achieving patient satisfaction [1,2,3,7,8]. However, the levels of job satisfaction for the nurses revealed inconsistent results for each study [9,10,11].

In measuring quality of health care, Donabedian’s three components (structure, process, and outcomes) approach is widely used [12]. He believed that structure measures have an effect on process measures, which in turn, affect outcome measures, but he also mentioned that cause and effect are more complex in the real world. On the basis of the Donabedian model, we postulated that our desired impact of ‘high-quality nursing care for cancer patients’ was determined by three different types of measures, namely structure, process, and outcome.

To date, academic research on the comprehensive nursing ward is mainly focused only on nursing-related fields. There are few studies currently available regarding the influence of comprehensive nursing applied to patients suffering from malignancies. As is well known, cancer patients require complex medical attention as they undergo surgery, chemotherapy, radiotherapy, and concurrent supportive care. The importance of offering sophisticated cancer patient-directed nursing, combined with anticancer treatments, to maximize patient survival outcomes and achieve a better quality of life, cannot be overemphasized. In this context, we adopted this new nursing system in one of the hematology-oncology wards (called cancer-oriented comprehensive nursing wards) to review its operational outcomes in comparison with those in the preexisting general cancer ward.

## 2. Materials and Methods

### 2.1. Study Setting

A tertiary hospital (827 beds) staffed with 246 doctors and 703 RNs is located on the east coast of the Republic of Korea. In 2018, 11,946 patients diagnosed with cancer were admitted to the hospital (average 32.4 patients/day, bed turnover rate 85.7%) and 5682 inpatients received chemotherapy in cancer care wards. Besides a preexisting general cancer ward (58 beds with 25 RNs only), we started to run a new cancer-oriented comprehensive nursing ward (46 beds with 25 RNs and 12 NAs) in December 2017. These two wards mainly cared for medical hematology-oncology patients. A total of 11,946 inpatients (average 32.4 patients/day, bed turnover rate 85.7%) were admitted, and 5682 patients received chemotherapy in 2018.

### 2.2. Participant Selection and Data Collection

Respondents (patients and nurses) who had consented to participate were enrolled in this preliminary study. First, patients who met the following criteria were eligible: (a) diagnosed with cancer, (b) community-dwelling, and (c) without any missing data. We determined the patient sample size using G*Power version 3.1.9.2. The sample size was calculated based on the effect size of 0.5, a significance level of 0.05, and a power of 0.80, and the minimum sample size was 52 in each group. From March 2019 to February 2020, data were collected for 104 patients (52 patients in the comprehensive ward and 52 in the general ward, respectively). A total of 102 patients were ultimately analyzed (two patients in the general ward submitted incomplete questionnaire form). Second, 42 nurses volunteered to participate in this study (23 nurses out of 25 in comprehensive ward and 19 out of 25 in general ward, respectively). Each questionnaire form (either for patients or for nurses) informed respondents about the confidentiality of their answers and the voluntary nature of their participation. Additionally, the purpose and method of the study were clearly specified and consent for participation was obtained. Participants could always stop the survey at by their own choice, without any penalty.

### 2.3. Measurements

**Figure 1:**
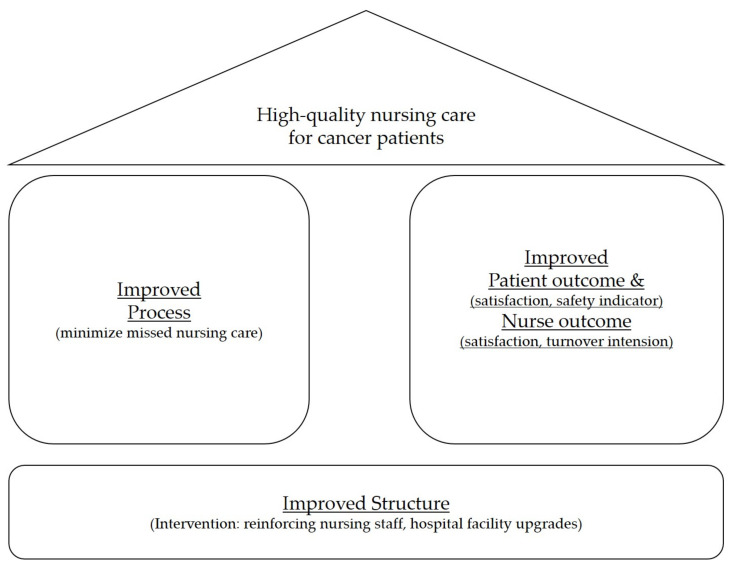
Conceptual framework of our study.

In order to assess the quality of nursing services with simple but consistent variables, we adopted the Donabedian model for examining the quality of care to analyze our clinical outcomes regarding the cancer-focused comprehensive nursing service in three main categories: structure, process, and patient/nurse outcomes (Figure 1) [1,12].

#### 2.3.1. Structure

At first, we looked into structural improvements made in the cancer-oriented comprehensive nursing ward. Changes in the number of nursing staff, the nurse-patient ratio, and any hospital facility upgrades were documented.

#### 2.3.2. Process

The indicator of the process category included any item of missed nursing care (or care left undone) in our study. Nursing care indicates (a) communication and information sharing; (b) education, including care planning, discharge planning, and decisions; (c) fundamental physical care; and (d) emotional and psychological care. Missed nursing care encompasses any unfinished or delayed clinical, administrative, or emotional care that was not completed or was postponed during a nurse’s given duty time [13]. The reporting and tracking of missed nursing care in the study population was performed by a nurse in charge, under the supervision of head nurse, on a monthly basis.

#### 2.3.3. Patient/Nurse Outcome

Patient satisfaction and safety indicators, such as falls and infections, were assessed as patient outcomes. Using the patient satisfaction index [2], a questionnaire comprised of 35 questions (on a 5-point Likert scale) was utilized to compare patient satisfaction between the comprehensive nursing ward and the general ward. The patient satisfaction in physical, therapeutic, environmental, emotional, and informative nursing was included. A higher score in the survey indicated a higher level of patient satisfaction. Moreover, we additionally asked patients whether they wanted to re-visit the comprehensive ward based on the followings: “If there is a need to admit to the hospital, would you want to use comprehensive nursing ward again?” and “Are you willing to encourage other patients to try this ward?” [14,15]

In terms of nurse outcome, job satisfaction and turnover intention (or will to resign) were evaluated. First, a well-designed tool to measure nurses’ job satisfaction was adopted in our study, which was composed of 20 questions (assessed on the 5-point Likert scale) [16,17,18]. The higher the score, the higher the job satisfaction. Questionnaire items included satisfaction with the professional position, payment level, interaction, autonomy, job requirements, administrative affairs, and the relationship between nurses and other healthcare professionals. Second, turnover intention was measured using 6 questions (on a 5-point Likert scale) [19,20]. A higher score indicated a nurse’s higher will to resign. In order to guarantee anonymity and to obtain accurate answers, we decided not to ask nurses for personal data, such as age, education level, and service period.

### 2.4. Statistical Analysis

When comparing the characteristics of patients in two different groups, the Chi-square test and the Fisher exact test were used for categorical variables, and the Student’s t-test and the Mann–Whitney U test were used for continuous variables. For the analysis of the study’s main outcome (comparison of satisfaction between the two wards), we used the Student’s t-test with the score results from each group. The statistical significance referred to a value of *p* < 0.05. Data were analyzed using IBM SPSS version 24.0 for Windows (SPSS Inc., Chicago, IL, USA).

## 3. Results

### 3.1. Structure

Structural (nursing staffing and environment) upgrades were made in the cancer-oriented comprehensive nursing ward. The nursing staff was reinforced in this special ward (46 beds with 25 RNs and 12 NAs) when compared with the general ward (58 beds with only 25 RNs). This finally resulted in the improved nurse-patient ratio (1:8 in the comprehensive ward as compared with 1:14 in the general ward).

Environmental upgrades were made to promote patient safety, including a wider space between beds (1.5 m vs. 1.0 m), fully automatic beds with fall prevention sensors, etc. Mobile toilets, shampoo aids, a sink, and room shower equipment are available for patients to perform activities of daily living, with the help of circulating NAs. Furthermore, auxiliary nurse stations were arranged by the corridor to further support nurses’ work, and closed-circuit televisions were also installed to monitor any safety risks, such as falls (Table 1).

### 3.2. Process

**Figure 2:**
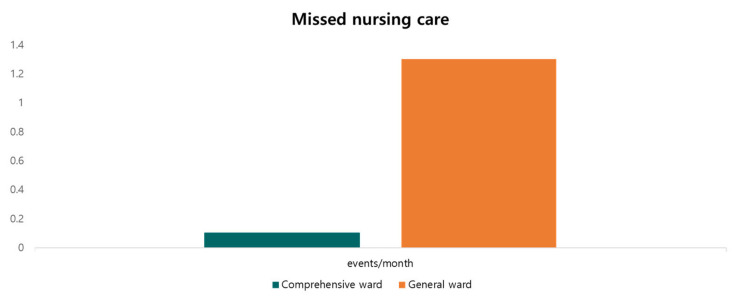
Missed nursing care (events/month).

We examined missed nursing care as an indicator of the process category. From March to November 2019, the incidence of missed delivery nursing care was only one in the comprehensive ward, as opposed to twelve in the general ward (an average 0.1 and 1.3 events per month, respectively) (Figure 2).

### 3.3. Outcomes

#### 3.3.1. Patient Outcome

A survey regarding the patient outcome in both groups was performed by the time the patient was discharged. The mean age of patients was similar in between groups (62.38 ± 17.67 years and 62.62 ± 10.22 years in the comprehensive ward and the general ward, respectively). More than 60% were male (61.5% and 60%, respectively) and the Eastern Cooperative Oncology Group performance status was either 0 or 1 in most of the patients in both wards (82.7% and 76%, respectively). Colorectal cancer patients accounted for the largest proportion (28.9% and 32.0%, respectively) in our study. The differences in the nursing requirement severity between the two wards were not statistically significant (*p* = 0.06). The detailed baseline characteristics of inpatients with solid or hematologic malignancies are summarized in Table 2.

**Figure 3:**
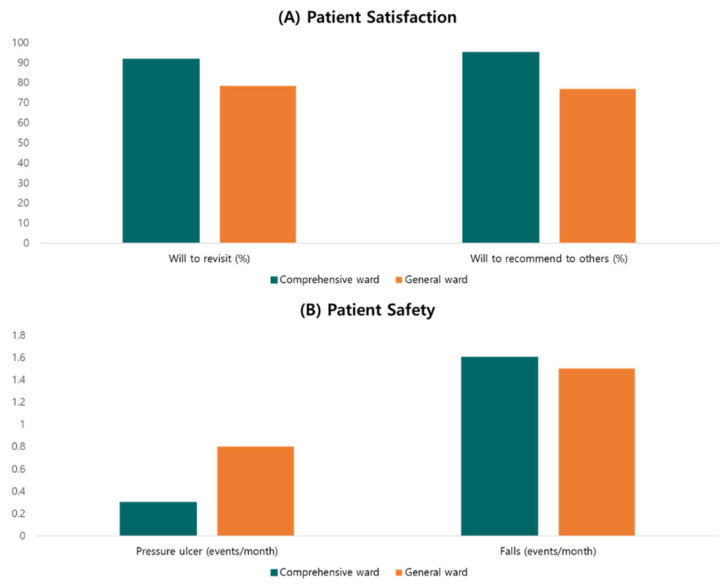
(**A**) Patient satisfaction (willing to re-visit and recommend to others, %); (**B**) patient safety (pressure ulcers and falls, events/month).

Patient satisfaction was measured by the patient satisfaction index. The scores of all five items (i.e., physical, therapeutic, environmental, emotional, and informational nursing satisfaction) were statistically higher in the comprehensive ward than those in the general ward (Table 3). We also conducted an additional survey on patients’ willingness to revisit and willingness to recommend to others (willing to revisit: 91.7% vs. 78.4%, willing to recommend to others: 95.0% vs. 76.8%) (Figure 3A). In terms of patient safety, the incidence of pressure ulcers was improved in the comprehensive ward (0.3 event/month in the comprehensive ward and 0.8 events/month in the general ward). However, there was no difference in the incidence of falls between the two wards (1.6 event/month vs. 1.5 event/month) (Figure 3B).

#### 3.3.2. Nurse Outcome

The nurse outcome survey was conducted simultaneously three months after the start of the study in both groups. The overall job satisfaction of nurses was found to be better in the comprehensive ward (M ± SD; 3.54 ± 0.33) than that in the general ward (M ± SD; 3.09 ± 0.55). We also found that a nurse’s intention to resign (or reposition) was more stable in the comprehensive ward than that in the general ward (M ± SD; 3.10 ± 0.51 vs. 3.87 ± 0.49, *p* < 0.01) (Table 4).

## 4. Discussion

In this study, we evaluated new comprehensive nursing care services in view of the structure, process, and patient/nurse outcomes. We compared not only subjective items (patient and nurse satisfaction) but also objective items, such as falls, pressure ulcers, and missed nursing care errors. Innovative changes were identifiable in all the subjective and objective variables (except for the incidence of falls), which are essential to maintain the high standard of cancer treatment.

In Western countries, the nursing care delivery system has evolved to a nurse-centered scheme which strictly restricts non-medical personnel from staying with patients to help with medical care. On the other hand, due to a lack of medical staff (mainly doctors and nurses) and equipment, some of the healthcare provider’s duties are still passed on to the family members or paid caregivers in our country. This outdated nursing system really can be potentially harmful to cancer patients because anticancer treatment requires the multidisciplinary collaboration of healthcare workers, as well as an enormous amount of medical resources [21]. We aimed to estimate the performance outcomes of a cancer-centered comprehensive nursing ward (which features nursing staff reinforcement and hospital environment upgrades), in comparison with the current general cancer ward as a new way of hematology-oncology patient nursing care.

The nursing staff is crucial in delivering the best available medical care for cancer patients [22]. To improve their quality of life, it is very important to simultaneously provide high-quality nursing services and anticancer treatment. In this respect, we postulated that a comprehensive nursing system could provide us with good insight into ideal inpatient services for cancer patients. It can also relieve the financial burden on cancer patients and their family members. Patients with malignancy, especially advanced or metastatic, often have difficulty performing routine daily activities, so their need for caregiving services is relatively higher than those of non-cancer patients. Under this new system, with the help of a national insurance subsidy, cancer patients only need to pay a quarter of the caregiving fee instead of hiring a private caregiver when they use the comprehensive nursing services [1].

In this study, we conducted a pilot study, for the first time in a literature review, on the operational performance of a cancer-focused comprehensive ward. With reference to previous studies [1,2], we chose simple but clinically useful parameters from each category (structure, process, and outcome) in regards to an oncologist’s perspective. In the structure, the most significant changes were made in the software aspect (the number of RNs and NAs was increased to achieve a nurse-patient ratio of 1:8) in addition to hardware aspects (facility upgrades). In the general ward, one duty nurse takes charge of fourteen patients, i.e., cancer patients were put at great risk of exposure to poorer quality medical care, even if this was not the intention of the system. We further reinforced the system with seven NAs to provide the patients with meal, bathing, personal hygiene, and toileting assistance. Thus, the RNs were able to invest more time in their profession-related tasks, such as patient education, occupational training, etc. We determined that a comprehensive nursing system can help to maintain a desirable nurse-patient ratio so as to create a healthier work environment for both patients and medical staff [23]. As stated above, we performed many renovations to hospital environments to ensure patient safety and comfort during the hospital stay. In the near future, we hope that environmental improvement in the general ward can also be ensured through the use of government support programs.

A previous study proposed nursing time, missed nursing care, and service quality as process outcomes [1]. We selected missed care because it is the single most important representative of process outcomes, as it is easy to monitor and share among doctors, RNs, NAs, and other healthcare providers. We observed that care left undone is dramatically reduced in the comprehensive nursing ward.

Many studies demonstrated that patient satisfaction and hospital reuse intention are consistently higher in comprehensive nursing wards [1,24]. We also observed the same result, with statistical differences, in the cancer patients using the comprehensive service. We also evaluated whether the cancer-focused comprehensive ward could be helpful in securing patient safety because cancer patients are vulnerable to pressure ulcers and pathologic fractures from falls. Interestingly, we observed that the incidence of falls was similarly low between the two groups. This might be partly explained by our baseline characteristics of relatively young age and good performance status in our study.

When the nursing staff is satisfied with their jobs, we can expect that a higher quality of nursing services can be ensured. This is why it is vital to take a close look at nursing outcomes when evaluating a cancer-directed comprehensive nursing care. In the literature review, studies have thus far shown mixed results concerning this topic. Some studies reported higher nurse satisfaction [25]; others showed a lack of statistical difference [23]; while another revealed lower nurse satisfaction [26]. In our study, job satisfaction was higher in the comprehensive ward than in the general ward. However, there were a few things to consider: although the differences in nursing severity were not statistically significant, nursing severity in the general ward patients was somewhat higher. Moreover, experienced nurses were assigned in the early stages of the comprehensive ward operation. Therefore, care must be taken in interpreting our job satisfaction results. Based on these findings, we must closely monitor the nursing staff and equipment to maintain a higher level of patient/nurse outcomes [27,28], which secures professional care for cancer patients.

There are some limitations in our study. First, our findings originated from a preliminary, non-randomized study with a small sample size that was not representative of the larger population. Second, the purpose and method of the research were clearly communicated to the participants, and the non-blinded nature of study design could definitely result in inevitable statistical bias; there was evident superiority and inferiority in our variables between the two groups, including clear differences in manpower and resources. Third, the self-reporting form of the questionnaire was the main source of information to evaluate the quality of nursing care. Fourth, among the various nurse-related outcomes, only job satisfaction and turnover intention were included. In patient-related outcomes, only patient satisfaction, ulcer sores, and incidental falls were analyzed. Further studies are needed to encompass other important aspects of medical care, such as opportunistic infection rates, length of hospital stay, thromboembolic events, and survival outcomes.

## 5. Conclusions

Our study was a pilot study to demonstrate that a cancer-oriented comprehensive nursing service would be successful both in improving the quality of cancer care and the nursing staff’s job satisfaction. We believe that our experiences are not confined only to medical hematology-oncologists or cancer nurses in the Republic of Korea. In other words, to achieve and maintain high-quality cancer patient care, continued interest and efforts to improve the nursing care delivery system are also crucial.

## Figures and Tables

**Table 1:** The environmental improvements in the comprehensive nursing ward.

Category	Facilities and Equipment			Purpose
		General Ward	Comprehensive Ward	
Ward	Auxiliary nurse station per team	None	A table with one PC * per team	Proximity nursing
	Patient lounge	Shared and small	More spacious	Providing comfortable space
	Corridor surveillance cameras	Same		Incident/accident monitoring
	Corridor guard rail	In compliance with government regulations	Twice as protective as required per the regulations	Fall prevention
Patient room	Interval between beds	1.5 m	2 m	Infection prevention
	Emergency call bell per bed	Same		Fall prevention
	Mat with fall prevention sensor	None	Yes	
	Electric bed with remote control	Beds in private rooms only	All of the beds	Patient convenience
	Air mattress	None	Yes	Bedsore prevention
	Sink per room	Same		Infection prevention
	Alcohol-rub dispenser per bed	Same		
	Toilet in room	Beds in OB/GYN ** ward and private rooms	About 30% of beds	Patient convenience
	Shower room	Beds in private rooms only	About 30% of beds	
Medical equipment	Wheelchair, walker	In compliance with the government regulations	More than 1.5 times as many as required per the regulations	Fall prevention
	Mobile toilet	None	Yes	Sanitary nursing
	Shampoo aid	None	Yes	
	Bath bed	None	Yes	
	Oxygen monitor	In compliance with the government regulations	Twice as many as required per the regulations	Vital sign measurement
	Non-invasive sphygmomanometer	In compliance with the government regulations	Twice as many as required per the regulations	
	Bladder scan	Same		Residual urine check
	Various and detailed patient information	Handout only	Handout and video guide	Educating patients and visiting guardians

* PC, personal computer; ** OB/GYN, obstetrics and gynecology.

**Table 2:** Baseline characteristics of patients (*n* = 102).

Characteristic	Comprehensive Nursing Ward (*n* = 52)	General Ward (*n* = 50)	*p*
	*n* (%) or M ± SD *	*n* (%) or M ± SD	
Age (years, range)	62.38 ± 11.17 (19–86)	62.62 ± 10.22 (38–84)	0.84
Sex			
Male	32 (61.5%)	30 (60%)	0.87
Female	20 (38.5%)	20 (40%)	
Living status			
Living with family	27 (75%)	34 (82.9%)	0.39
Living alone	9 (25%)	7 (17.1%)	
ECOG PS **			
0–1	43 (82.7%)	38 (76%)	0.32
2	5 (9.6%)	6 (12%)	
3	3 (5.8%)	2 (4%)	
4	1 (1.9%)	4 (8%)	
Length of stay (days, range)	9.98 ± 4.51 (2–29)	10.50 ± 6.40 (3–33)	0.56
Reason for admission			0.33
Elective	44 (88%)	39 (78%)	
Emergency	8 (12%)	11 (22%)	
Cancer stage (solid tumor)			
I-II	10 (25.0%)	7 (14.6%)	0.22
III-IV	30 (75.0%)	41 (85.4%)	
Cancer type			0.78
Breast	12 (23.1%)	9 (18.0%)	
Colorectal	15 (28.9%)	16 (32.0%)	
Hepatobiliary/Pancreatic	3 (5.8%)	4 (8.0%)	
Lung	6 (11.5%)	6 (12.0%)	
Stomach	2 (3.9%)	5 (10.0%)	
Hematologic malignancies	13 (25.0%)	7 (14.0%)	
Others	1 (1.8%)	3 (6.0%)	
Nursing requirement severity			
I-II	51 (98.1%)	44 (88%)	0.06
III-IV	1 (1.9%)	6 (12%)	

* M ± SD, mean ± standard deviation; ** ECOG PS, Eastern Cooperative Oncology Group performance status.

**Table 3:** Scores using the patient satisfaction index (*n* = 102).

Characteristic	Comprehensive Nursing Ward (*n* = 52)	General Ward (*n* = 50)	*p*
	Score *, M ± SD **	Score, M ± SD	
Physical satisfaction	4.32 ± 0.66	4.02 ± 0.71	<0.01
Therapeutic satisfaction	4.33 ± 0.69	4.09 ± 0.66	<0.01
Environmental satisfaction	4.41 ± 0.70	3.95 ± 0.77	<0.01
Emotional satisfaction	4.39 ± 0.66	3.99 ± 0.75	<0.01
Informational satisfaction	4.29 ± 0.69	3.97 ± 0.88	<0.01

* The scores range from 1 to 5; ** M ± SD, mean ± standard deviation.

**Table 4:** Comparison of nurses’ job satisfaction and turnover intention (*n* = 42).

Characteristic	Comprehensive Nursing Ward (*n* = 23)	General Ward (*n* = 19)	*p*
	Score *, M ± SD **	Score, M ± SD	
Job satisfaction	3.54 ± 0.33	3.09 ± 0.55	<0.01
Turnover intention	3.10 ± 0.51	3.87 ± 0.49	<0.01

* The score ranges from 1 to 5; ** M ± SD, mean ± standard deviation.

## Data Availability

Our data are readily available upon reasonable request.
